# Derivation of ES-like cell from neonatal mouse testis cells in autologous sertoli cells co-culture system

**Published:** 2014-01

**Authors:** Mohammad Hossein Asadi, Setareh Javanmardi, Mansoureh Movahedin

**Affiliations:** 1 *Department of Anatomical Sciences, Faculty of Medical Sciences, Baqiyatallah University of Medical Sciences, Tehran, Iran.*; 2 *Department of Anatomical Sciences, Medical Sciences Faculty, Tarbiat Modares University, Tehran, Iran.*

**Keywords:** *Spermatogonial stem cells*, *Embryonic stem like cell*, *Pluripotent stem cells*, *Co-culture*

## Abstract

**Background:** Spermatogonial stem cell (SSC) is a self-renewing population of male adult stem cell. SSCs have a differentiation potential which are similar to embryonic stem cells. These Embryonic stem like (ES-like) cells can be a potential source for pluripotent cells for stem cell-based therapy.

**Objective: **This study presents an economical and simple co-culture system for pluripotent stem cells generation from neonatal mouse testis

**Materials and Methods:** Isolated testicular cells were cultured in DMEM/F12. Characteristics of the isolated cells and obtained ES-like cell were immune-cytochemically confirmed by examining the presence of PLZF, vimentin, Oct4 and Nanog protein. Expression of the pluripotency and germ-cell specific genes was analyzed by qPCR in derived ES-like colony and SSCs respectively.

**Results:** The experiment results indicated that our method of obtaining pluripotent ES-like cells from spermatogonial cells (SCs) is simpler than the described methods. ES-like cells were immunopositive for pluripotency markers. ES-like cell qPCR results indicated signiﬁcant increase in pluripotency genes expression and signiﬁcant decrease in germ cell-speciﬁc genes expression.

**Conclusion:** The results indicated that ES-like cell with pluripotency characteristic were generated from freshly isolated spermatogonial cells. The pluripotent stem cells provide a cellular reservoir usable for regenerative medicine instead of embryonic stem cells.

This article extracted from Ph.D. thesis. (Setareh Javanmardi)

## Introduction

Spermatogonial stem cells (SDCs) are part of a subset of male germ cells which are called undifferentiated spermatogonia (1). Although the most important role of SSCs is the unlimited production of sperm, they are the unique population of male adult stem cells which have the responsibility of passing genetic material to the next generation (2-4). In the testicular microenvironment, SSCs produce only the spermatogenic lineage. However, several studies demonstrated that when SSCs are isolated from the testis and placed into a specific environment for extended periods, they proliferate and form three-dimensional spermatogonial aggregations (colony) and acquire embryonic stem cell properties (5-10). Pluripotency of these ES-like cells has been demonstrated by their capacity to contribute to germ-line chimaeras and differentiation into tissues which belong to all three embryonic germ layers. ES-like cells express a blend of both germ line and embryonic stem cell markers (9, 11-13). 

This property may allow individual cell-based therapy without the ethical and immunological problems associated with human embryonic stem cells (14, 15). In previous studies, pluripotent stem cell lines were generated from SSCs derived from neonatal mouse testis as well as from adult mouse and human testis (6, 9, 11). Some experiments have shown that SSCs indefinitely self-renewed and robustly proliferated in the presence of feeder layer and growth factors such as glial cell line-derived neurotropic factor (GDNF) and fibroblast growth factor (FGF2) (16, 17). Guan *et al* did not use the distinct stem cell medium described by Kanatsu-Shinohara *et al* but used Dulbecco's Modified Eagle Medium (DMEM) with serum and added GDNF, where testicular cells were initially cultured for 4-7 days (9, 11). 

All these groups added GDNF and growth factors to the culture, either continuously or initially (9, 11, 14). GDNF, a distant member of the transforming growth factor beta (TGF-β) family, which controls SSC self-renewal is critical for the maintenance of permanent spermatogenesis (18, 19). In vivo GDNF and several growth factors were produced by sertoli cells, which are crucial component of SSC microenvironment. These cells support the germ cell development by providing structural support, secreting necessary cytokines and growth factors (20-23). Although several methods of sertoli cells and SSCs co-culture are reported, in most of them sertoli cells of different source or cell line were served as feeder layer or in vitro condition the development of SSCs were observed for a short time (24-28). 

Therefore, we used co-culture system to supply the critical factors from sertoli cells as well as SCs instead of adding any growth factors or different source of sertoli cells for SCs derived ES-like cells. In summary, we described the derivation of pluripotent stem cells from the neonatal mouse testis by using simple culture condition.

## Materials and methods

**Testicular cell isolation and culture**

In this experimental (in vitro) study protocols for animal care and surgical intervention of neonate mouse in this study were approved by the Institution Animal Care and Use Committee at the University of Baqiyatallah, Tehran, Iran. In this study bilateral testis was collected aseptically from newborn (0-2 days old) NMRI mice. Testicular cells were collected by two-step enzymatic digestion using collagenase and trypsin (27). 

Viability of collected cells was analyzed by trypan blue dye. We used a co-culture system for expansion of spermatogonial cells and ES-like cells generation. In this culture system the testicular cell suspension which included mainly spermatogonia and sertoli cells were incubated together in DMEM/F12 supplemented with 100 IU/ml penicillin 100 μg/ml streptomycin, and 40 μg/ml gentamycin (all from Invitrogen, USA), single-strength non-essential amino acid solution (Gibco, Invitrogen), 10% fetal bovine serum (FBS), 1 mM L-glutamine (Invitrogen), 0.1 mM b-mercaptoethanol (Sigma) and 1,000 units/ml Leukemia Inhibitory Factor (LIF; Sigma, USA). Medium was changed every 3 days, and all cultures were passaged every 5 days with trypsinization and subculture at a one-half to one-third dilution depending on their proliferation state. 

All cultures were maintained at 37 in a 95% humidified atmosphere of 5% CO_2_. Cells were analyzed microscopically every day. Spermatogonial cells were purified by differential plating. For molecular confirmation of ES-like cells generation (RT-qPCR, Immunocytochemistry) DAZL, Piwill2, Stra8 and Mvh expression level in freshly isolated spermatogonia cells were compared to those in ES-like cell colonies. 

**Embryoid Body generation**

Cultured cells were passed and propagated to ES-like cell colonies generation for 3 weeks. These colonies could be observed by a microscope and could be scraped off using a pipette tip which will minimize, if not eliminate, the contamination of non-ES like cells. Therefore, these colonies were separated mechanically and were triturated to a single-cell suspension before they were transferred to uncoated 6-cm no adherent bacteriological petri dishes (Greiner) in previous medium supplemented with 5% FBS and without LIF to allow for embryoid body (EB) formation.

**RNA extraction and real-time PCR analysis**

Total cellular RNA from SCs-derived ES-like cells, isolated SCs and mouse ES cells were extracted with the RNeasy Micro kit (Qiagen, USA) according to the manufacturer’s instructions. Concentrations of RNA were determined by UV spectrophotometry (Eppendorff, Germany). DNase treatment was performed to clean up RNA possible genomic DNA contamination. One µg of total RNA was then transcribed to cDNA using the Quantitect RT kit (Qiagen, USA). 

PCR ampliﬁcation was performed using gene-speciﬁc forward and reverse primers (Table I) as Follows: an initial melting cycle for 5 min at 95^o^C to activate the polymerase, followed by 35 cycles of melting (30 sec at 95^o^C), annealing (30 sec at a speciﬁc annealing temperature for each primer which are shown in Table I) and extension (30 sec at 72^o^C). For QRT-PCR, 5 ng of cDNA template was used in a 25 µl reaction volume with Quantitect Sybr Green PCR master mix (Qiagen, USA) and run on a BioRad iCycler. The quality of the PCR reactions was confirmed by melting curve analyses. The efﬁciency of each gene was determined by a standard curve (the logarithmic dilution series of testis cDNA). 

The Comparative CT Method (2^ΔΔCT^) was used to determine the relative quantification of target genes, normalized to a housekeeping gene (GAPDH or βactin) and related to a calibrator (Embryonic stem cells). The validity of the experiment was performed to verify that the target efficiencies and reference were approximately equal. The RT-PCR analysis of at least three independent cultures was performed for all experiments. RNA from embryonic stem cell (CGR8 line), was used as positive control and negative control was mock reverse transcription with DEPC-treated water instead of RNA or cDNA samples. 

**Immunocytochemistry**

To characterize, we washed spermatogonia, sertoli and SCs-derived ES-like cells with PBS and fixed in 4% paraformaldehyde (PFA) in PBS (pH 7.4) for 20 minutes. Then cells were washed twice with PBS/0.1% Tween-20 to remove residual fixative. At the next step, Permeabilization was performed by 4% Triton 100X (Sigma) in PBS for 30 minutes. Non-specific binding sites were blocked by incubation of cells with 10% serum of secondary antibody species in PBS for 30 minutes. Procedure followed by incubation with antibody solution overnight at 4^o^C. Primary antibodies consisted of: Oct4 (1:500; unconjugated anti-Oct4, Abcam), Nanog (1:200; rabbit polyclonal anti- Nanog, Abcam), PLZF (1:300; rabbit anti-PLZF, Abcam), Vimentin (1:100; rabbit polyclonal anti-Vimentin). 

The next day, cells were washed twice with PBS/0.1% Tween-20, 5 minutes and appropriate secondary antibody, FITC conjugated anti-rabbit IgG (1:5oo, Abcam) and Texas Red anti-Rabbit IgG (1:300, Abcam) were used. The cells were washed three times for 5 min with PBS to remove unbound IgG and dye. Then nuclei were counterstained with 4, 6-diamidino-2-phenylindole (DAPI) (Roche, Switzerland). Images were captured with a Fluorescence microscope (BX51, Olympus, Tokyo, Japan). Neonatal mouse testis and embryonic stem cells (P19 line) were used as positive control (Figure 4). For negative controls, equivalent concentrations of normal immunoglobulin of the primary species were used instead of primary antibodies. 

**Alkaline phosphatase activity assay**

On the 20^th^ day or passage 4, ALP activity was determined in ES-like colonies by a commercially available alkaline phosphatase kit (Sigma). Firstly, the medium was removed and the colonies were washed three times with Tris Buffered Saline (TBS). Secondly, the fixation was performed with 10% formaldehyde at 4^o^C for 10 minute. Thirdly, colonies were rinsed twice with 0/2 M Tris buffer (pH=8.9) and activity was represented by freshly prepared substrate reagent (0.01% naphtol-AS-MX phosphate and 0.06% fast violet salt in 0.1 M Tris buffer, pH=8.9) which was released after incubation at 37^o^C for 30 minutes. Finally, colonies were washed with dH_2_O and were mounted. Colonies with dark-red color were considered positive for ALP activity.)

**Statistical analysis**

The obtained data was analyzed through SPSS 16.0 software as well as the independent-samples Student’s *t*-test. Data are expressed as mean±SD. P-value<0.05 was defined as statistical significance.

## Results

**Morphological analysis of ES-like cell colony**

**Figure 1:**
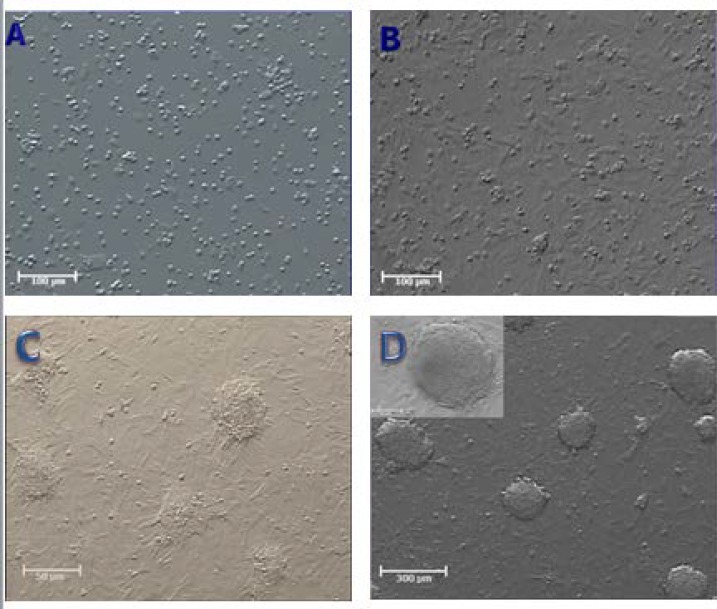
Morphology and characterization of primary cell cultures and colony formation of mouse neonate testis (A) Cell population obtained from the seminiferous tubules (B) morphology of primary neonate mouse testis cells, cultured in free-growths factors DMEM/F12, 24 h after primary culture (C) Colonies of spermatogonial stem cells (SSCs) after 5 days of culture (D) Colonies of spermatogonial stem cells (SSCs) after 2 weeks of culture

After enzymatic dispersion, we obtained a SCs/somatic-cell mixture. Most testis cells were attached to the growing surface two days of postplating. These cells proliferated and created a monolayer of cells. Uniquely developed spermatogonial cells shaped colonies after approximately 5 days which were characterized by a morula-like structure. SCs proliferated logarithmically over a 3 weeks period (Figure 1). 

**Figure 2:**
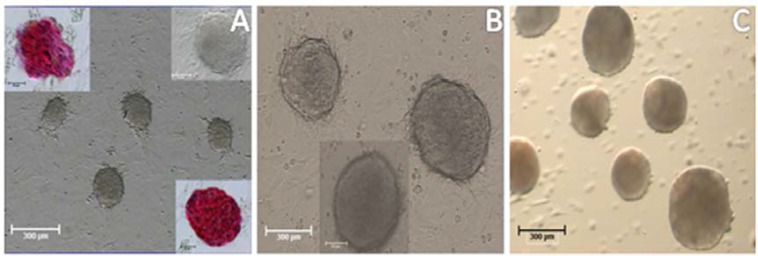
(A) Colonies of mouse neonate spermatogonial stem cell after 3 weeks of culture and the cell of these colonies showed positive alkaline phosphatase reactivity (B) An embryonic stem cell (ES)-like colony after 3 weeks of culture with sharp edged and compact appearance (C) Embryoid body structures generated from ES-like cells after 4 days culture on nonadherent plate

At end of the 3^rd^ week ES-like cells colonies were observed. ES-like colonies were sharply edged with compacted boundaries compared to SCs clusters which were appeared as clumps of individually visible cells (Figure 2). The number of ES-like colonies increased with time prolongation. One of the characteristics of pluripotent ES like cells is the ability of in vitro EB formation. Herein, EB structures were identified after 2 days of culturing on non-adherent plate (Figure 2).

**RT–PCR and Immunocytochemical analysis**

**Figure 3:**
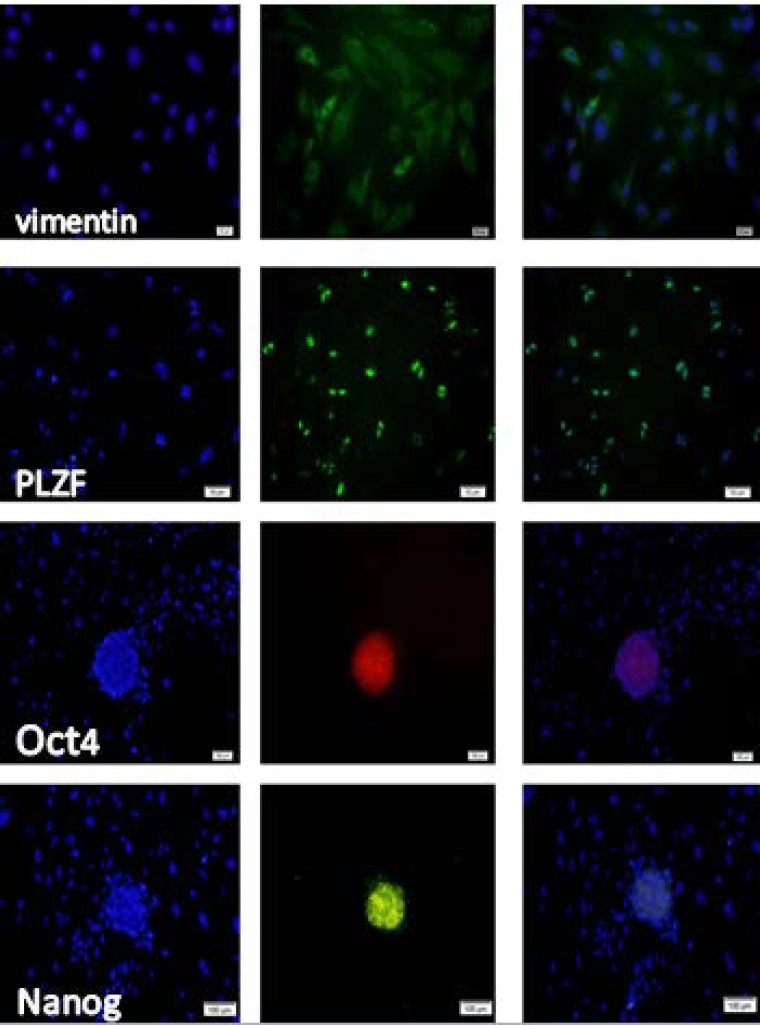
Immunofluorescence staining of neonate mouse sertoli cell (vimentin), Spermatogonial cell (PLZF) and SSCs derived ES-like colonies (Oct4 and Nanog).The left panel shows DAPI staining and the right panel shows the merged images

**Figure 4:**
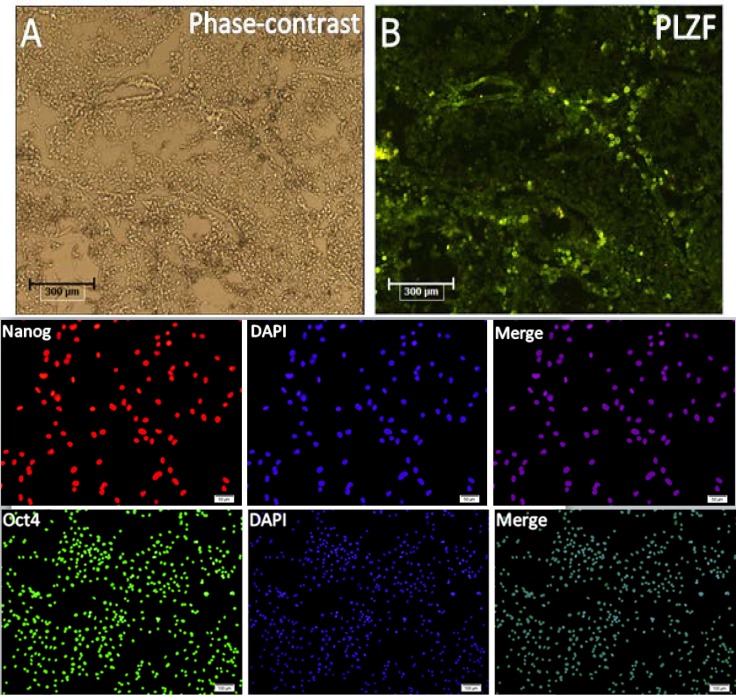
Immunofluorescence staining of neonate mouse testis as positive control for PLZF (A&B). Immunofluorescence staining of mouse embryonic carcinoma cell lines (P19 cells) as positive control for Nanog and Oct4

**Figure 5:**
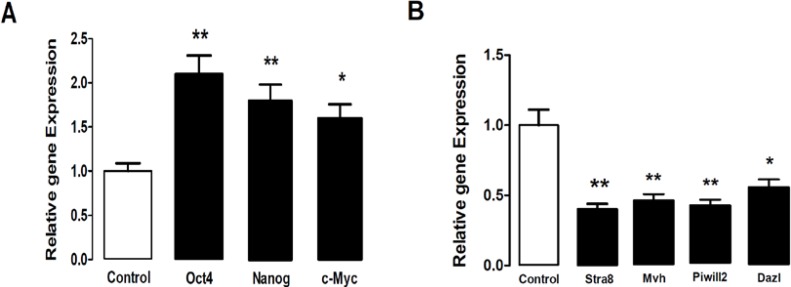
The ratio of pluripotency genes expression: Oct4, Nanog, c-Myc and Specific genes of germ cells: Stra8, DAZL, Mvh and Piwill2 in ES-Like cells relative to spermatogonial cells genes expression. The amount of spermatogonial genes expression was used as gene expression calibrator (Control). *p<0.05; ** p<0.01

**Figure 6:**
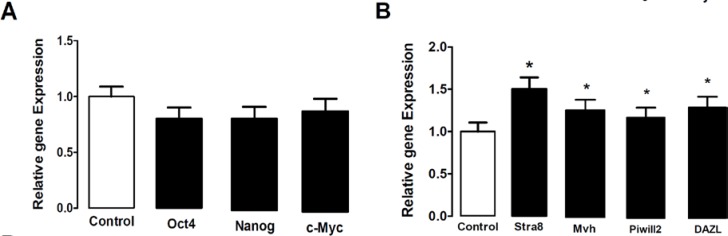
The ratio of pluripotency genes expression: Oct4, Nanog, c-Myc and Specific genes of germ cells: Stra8, Mvh, DAZL and piwill2 in ES- Like cells relative to Embryonic stem cells genes expression. The amount of Embryonic stem cells genes expression was used as gene expression calibrator (Control). * p<0.05; ** p<0.01

Isolated sertoli and spermatogonia cells were immunopositive to vimentin and PLZF protein respectively .The obtained ES- like cell colony expressed the Nanog and Oct4 protein (Figure 3) which are expressed in undifferentiated mouse ES too (Figure 4). Moreover, these ES-like cells colonies were strongly positive for ALP, which is characteristic of ES (Figure 2). To confirm our immunostaining data, we analyzed these SCs and ES-like colonies using RT-PCR as well. Results showed expression of associated pluripotency markers, such as Oct4, Nanog and c-Myc, whereas SCs freshly isolated from the testes were negative for these markers except Oct4. Furthermore, the expression of germ cell markers, *DAZL, Piwill2, Stra8* and *Mvh*, was Down-regulated in the ES-like colonies compared to the mouse-isolated SCs. These results demonstrate a change in gene expression in the mouse ES-like cells during dedifferentiation (Figure 5 and 6).

**Table I:** Primers used for Real-Time PCR

**Gene**	**Primer Sequences**	**Size** **(bp)**	**Ta (** ^o^ **C)**	**Genbank Accession**
Oct4	F: TGTGGACCTCAGGTTGGACT	201	58	NM 013633.2
	R: CTTCTGCAGGGCTTTCATGT			
Nanog	F: 5ʹ-CTGCTCCGCTCCATAACTTC-3ʹ	97	58	NM_028016.2
	R: 5ʹ-GCTTCCAAATTCACCTCCAA-3ʹ			
C-Myc	F: 5ʹ-CCCTCAGTGGTCTTTCCCTAC-3ʹ	229	58	NM_001177353.1
	R: 5ʹ-CCACAGACACCACATCAATTTC-3ʹ			
Stra8	F: 5ʹ-CTCCTCCTCCACTCTGTTGC-3ʹ	135	47	NM_009292.1
	R: 5ʹ-GCGGCAGAGACAATAGGAAG-3ʹ			
DAZL	F: 5ʹ-AAGGCAAAATCATGCCAAAC-3ʹ	72	57	NM_001277863.1
	R: 5ʹ-TCCTGATTTCGGTTTCATCC-3ʹ			
Mvh	F: 5ʹ-CGGAGAGGAACCTGAAGC-3ʹ	161	58	NM_010029.2
	R: 5ʹ-CGCCAATATCTGATGAAGC-3ʹ			
Piwill2	F: 5ʹ-CCTCCAGCTCTGTCTCCAAC-3ʹ	144	59	NM_021308.1
	R: 5ʹ-CCTTGCTTGACCAAAAGCTC-3ʹ			
Gapdh	F: 5ʹ-AACTTTGGCATTGTGGAAGG-3ʹ	132	58	NM_008084.2
	R: 5ʹ-GGATGCAGGGATGATGTTCT-3ʹ			
β2m	F: 5ʹ-TGACCGGCCTGTATGCTATC-3ʹ	198	58	NM 009735.3
	R: 5ʹ-CACATGTCTCGATCCCAGTAG-3ʹ			

F: Forward

R: Reverse

bp: base pairs

Ta: Optimal Annealing Temperture

**Figure 7:**
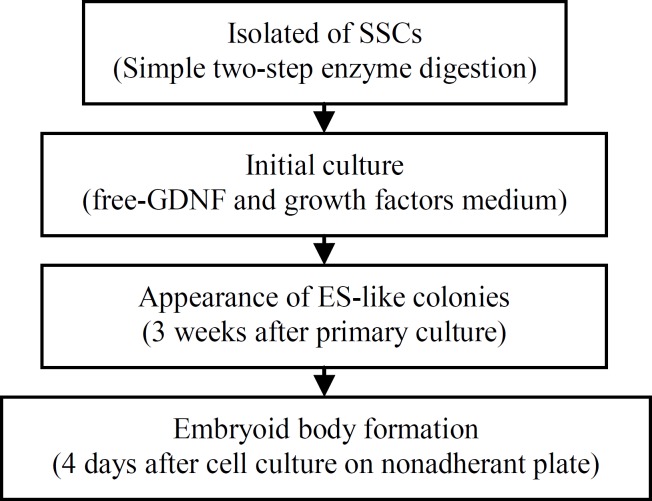
A summary of the procedures involved in obtaining ES-like cells from neonate mouse spermatogonial stem cell

## Discussion

Here we report the successful establishment of a simple and cost-effective co- culture system for ES-like cells derivation from neonatal mouse testis. Efficient derivation of ES-like cell colony from spermatogonial cell sources in vitro has been challenging in the treatment of cell therapy (29). spermatogonial stem cells can be genetically modified in vitro, and provide an alternative for embryonic stem cells in production of gene targeted mice after injection to infertile mouse testes (30, 31). On the other hand, several recent studies have demonstrated that SSCs can also revert back to pluripotency as embryonic stem like cells under certain culture conditions (17, 19, 30, 32, 33). 

With these valuable features, spermatogonial stem cell research holds great promises in not only the revolution of fertility preservation research, but also in clinical applications. SSCs cultures have been initiated from both prepubertal and adult testes, but the neonatal mice seem to provide the most optimal source as they have the relatively highest ratio of germ cells (16). Methods for culturing mouse and rat SSCs in vitro have been established in different laboratories (16, 31, 34). Indeed, a well-defined culture medium is required for optimal derivation and maintenance of the SSCs in undifferentiated conditions and to induce their genetic reprogramming toward ES-like colonies. In many studies, researchers have tried to provide natural condition such as microenvironment and growths factors for cell culture as far as possible. The previous studies have shown that ES-like cells arose in culture from neonatal testicular cells in the presence of GDNF and LIF on MEFs. These ES-like cells expressed ES cell speciﬁc markers (9). 

To generate ES-like colonies of this experiment, we used co-culture system because; it is assumed that GDNF and other necessary growth factors might be provided by the sertoli cells. In vivo SSC self-renewal and spermatogonial differentiation can be regulated by extrinsic factors from the somatic environment, and intrinsic genetic programs within germ cells. Sertoli cells are the only somatic cells that reside within the seminiferous tubules. These cells support the germ cell development by providing structural support, secreting cytokines such as immune protective factors, growth factors and nutritional factors of IGFI-1, BFGF, TGF, EGF, IL-1, IL-6, TNF, IFN-α, LIF, SCF, GDNF and transferrin (20-23).

These factors are necessary for SSC self-renewal and for directing fate decisions of all germ cells, including SSCs (35). Herein, we reported a step-wise and highly reproducible protocol to obtain ES-like cells by co-culturing the whole testicular cells in a selective culture system mimicking in vivo conditions. The difference between our study and the much published studies on SCs dedifferentiation to ES-like cells lies in the culture method used (6, 9, 11, 33). One of the most important factors that is secreted by sertoli cell is GDNF. This factor promoted the maintenance and proliferation of SSCs in vitro culture conditions. Although several methods of Sertoli cells and SSCs co-culturing are reported, in most of them Sertoli cells of different source were served as feeder layer (35-36). 

Recent studies have used Sertoli cell lines to support SSCs culture (24, 25). Due to having long term culture or isolation procedures for the establishment of cell line, the cells become transformed and lose some of their typical characteristics making them unsuitable for feeding SSCs (36). In this experiment, mixed cell including sertoli cells and SSCs were isolated of same age mouse and this coculture is used for 3 weeks. We observed the tightly packed embryonic stem cell (ESC)-like colonies after a mean of 3 weeks. These ES-like colonies were similar to the established mouse ES cells, not only in morphology but also in the expression of specific cell markers for pluripotent cells such as *Oct4, Nanog, Sox2 *(9, 11). 

These data indicated that the mouse ES-like colonies express the transcription factors which are the characteristic of undifferentiated ES cells. As described herein dissociated ES-like cells were able to aggregate and form EBs in suspension culture. Moreover, in previous studies, alkaline phosphatase reaction has been described as a marker for embryonic stem cells and our resulting ES-like colony showed alkaline phosphatase reactivity (11, 17, 30). 

Although ES-like cells can be stimulated by growth factors and other manipulation in vitro culture; however, to achieve intact ES-like cell without any epigenetic variations which can be used in clinic, the culture system must be similar to natural and in vivo condition with minimal physical and chemical intervention. In summary, in vitro condition we found the spontaneous generation of ES-like cells from neonate mouse testis under conditions known to support long term proliferation of SSCs (Figure 6). In addition, this experiment indicated that this defined and simple culture system provides significant practical advantages particularly for culture of SSCs and ES-like cells generation and studying mechanisms during the complex process of spermatogenesis and biological behavior of mouse SSCs that constitutes an important foundation for our future efforts. 

## Conclusion

Our results indicated that the freshly isolated spermatogonia could be reprogramed to pluripotent stem cells when they were cultured along with autologous Sertoli cells. The sertoli cells may provide growth factors and nutrient supplements which are critical for cell survival and expansion. It reduced consumption of additional growth factors and feeder layer that may cause undesirables genetic changes which might directed to mutation and cancer. In addition, the application of stem cells with no ethical problems and minimal intervention in vitro for regenerative medicine will be valuable. 
